# Adolescent Social Networks and Physical, Verbal, and Indirect Aggression in China: The Moderating Role of Gender

**DOI:** 10.3389/fpsyg.2020.00658

**Published:** 2020-03-30

**Authors:** Maoxin Zhang, Hongyun Liu, Yunyun Zhang

**Affiliations:** ^1^Faculty of Psychology, Beijing Normal University, Beijing, China; ^2^Centre for Educational Measurement, University of Oslo, Oslo, Norway; ^3^Beijing Key Laboratory of Applied Experimental Psychology, Faculty of Psychology, Beijing Normal University, Beijing, China; ^4^Collaborative Innovation Center of Assessment Toward Basic Education Quality, Beijing Normal University, Beijing, China

**Keywords:** aggressive behavior, physical aggression, verbal aggression, indirect aggression, friendship dynamics, gender differences, adolescents

## Abstract

Aggressive adolescents are preferable in some Western cultures, whereas Confucianism places great emphasis on the inhibition of aggressive behaviors in Chinese culture. Using the longitudinal social network analysis, we used a sample of 1354 Chinese adolescents (54% boys, ages 12–15) who were followed over 1 year at three time points to examine the association between friendship dynamics and physical, verbal, and indirect aggression and the moderating role of gender. This study found the following: (1) Students who were verbally aggressive were regarded as attractive, whereas those who were indirectly aggressive were unattractive as friends; (2) adolescents selected peers with similar levels of aggression as friends; (3) adolescents were influenced by their friends’ aggressive behaviors; and (4) girls were more susceptible than boys to the influence of physical aggression, although gender did not moderate the influence process of verbal and indirect aggression. The findings of this study provided a clearer insight into the selection and influence processes of the three subtypes of aggression and contributed to the diversity of samples. Chinese educators should pay more attention to both verbal aggression because of youths’ preference for it and to girls with physically aggressive friends since they are more susceptible than boys.

## Introduction

Aggression has been widely investigated by researchers regarding many aspects, such as different forms of aggression, gender differences, functions and impacts, risk factors, and culture-specific influences on aggression (e.g., Archer, 2004; Lansford, 2018). In recent decades, the associations between peers and aggression have been discussed particularly due to the salient role of peers in the development of aggression (Brechwald and Prinstein, 2011). As “behaviors that are intended to hurt or harm others” (Berkowitz, 1993), aggression has received much attention from parents, educators, and researchers. One of the primary reasons is that adolescent aggression is prevalent in nearly all countries and has a long-term impact on both aggressors (Ladd, 2005) and victims (Card et al., 2007), especially in predicting later antisocial behaviors and maladjustment (Card et al., 2008).

Particularly, the influence of peers is a key factor that affects adolescent aggression. It is documented that adolescents’ attitudes and behaviors are markedly similar to those of their friends (Brechwald and Prinstein, 2011). The homophily theory (Kandel, 1978) proposed that such similarities are attributable to youths’ initial preference to affiliate with friends who are similar to them (selection effects) and to the tendency to become more similar over time (influence or socialization effects). Exploring how teenagers acquire and develop different forms of aggressive behaviors in their social network (a representation of the relationships among a collection of individuals) lays an important foundation for effective prevention and intervention measures.

The first purpose of the current study is to examine the associations between adolescents’ social networks and the subtypes of aggression, providing a refined understanding of the homophily theory in adolescent aggression. Furthermore, whether gender plays a role in shaping peers’ aggression is the second question we aim to investigate. The existing studies regarding social networks and aggression mainly build on Western culture. However, cultural variation in aggression (Bergeron and Schneider, 2005) drives us to explore whether peers’ aggression has equal influences on adolescent aggression in Western culture and Eastern culture.

The remainder of this article is organized as follows. In the following part of this section, we review the literature regarding subtypes of aggression, friendship dynamics, and studies focusing on the relationships between them. Subsequently, we introduce our motivation, research questions, and hypotheses of the present study in detail. After describing the research methodologies, the results of this study will be presented. Finally, we conclude this article with a discussion.

### Subtypes of Aggression

There has been substantial research about aggression, but with an unbalanced emphasis on its three main subtypes – physical, verbal, and indirect aggression (Archer, 2004). Distinct definitions, developmental trajectory, impacts, and gender differences in the three forms of aggression are widely discussed (Björkqvist et al., 1992; Archer and Haigh, 1997; Archer, 2004; Card et al., 2008). Built on these differences in the varied forms of aggression, it is worthwhile and relevant to distinguish the subtypes of aggression, which motivated us to focus on physical, verbal, and indirect aggression individually in this study.

With regard to the definitions, physical aggression refers to physically assaultive behaviors, such as hitting or pushing; verbal aggression, as the name suggests, refers to verbal confrontations, such as threatening or making fun of others (Björkqvist et al., 1992). Physical aggression in addition to verbal aggression are thought to be direct forms of aggression. In contrast, indirect aggression is a more covert form of aggression in which the aggressor attempts to inflict hurt in such a manner that he or she seems not to intend (Björkqvist et al., 1992), such as ignoring, avoiding and excluding others.

Besides distinct manifestations, physical, verbal, and indirect aggression play dominant roles in different stages of youth (Björkqvist et al., 1992). In general, from childhood to adolescence, physical aggression declines, whereas verbal and indirect forms of aggression increase as teenagers gradually acquire verbal and social skills. The growing understanding of social norms also accounts for such transitions (Card et al., 2008), especially in cultures where physical aggression is severely unfavorable.

Furthermore, a meta-analysis study showed that the three subtypes of aggression have various impacts on adolescents (Card et al., 2008). For example, physical and verbal aggression have a stronger association with externalizing symptoms (e.g., delinquency), poor peer relationships, and low prosocial behavior, while indirect aggression is more related to internalizing problems and high prosocial behavior (Card et al., 2008). Verbal aggression, in particular, is rather toxic for cognitive reactivity, such as thinking ill thoughts about themselves (Cole et al., 2014).

Another primary research focus in aggression is gender differences. It is widely acknowledged that aggression processes vary between boys and girls (Lagerspetz et al., 1988; Archer, 2004; Tapper and Boulton, 2004). Particularly, it is well documented that physical aggression among boys is higher than that among girls, whereas gender differences in verbal and indirect aggression are much more ambiguous. Compared with girls, boys are more likely to adopt more overt aggressive behaviors (Card et al., 2008), such as kicking, striking, and shoving. Differences between boys and girls regarding physical strength (Björkqvist et al., 1992), masculine preference (Archer, 2004), instrumental and expressive beliefs in aggression (Archer and Haigh, 1997), and social expectations (Underwood, 2003) contribute to explaining such results. However, compared with physical aggression, research on gender differences in verbal aggression is less sufficient and coincident. The mixed results include that boys adopted less (e.g., Archer et al., 1988; Goldweber et al., 2013), equal (e.g., Lagerspetz et al., 1988; Gerlinger and Wo, 2016), or more (e.g., Toldos, 2005; Donoghue and Raia-Hawrylak, 2015) verbal attacks such as name-calling and arguing when comparing with girls. The inconsistent research results also apply to indirect aggression (Toldos, 2005; Wang et al., 2009; Gerlinger and Wo, 2016). Theoretically, indirect aggression is expected to be exhibited more frequently among girls. On one hand, girls develop verbal and social skills more quickly than boys, making it earlier for girls to develop indirectly aggressive strategies (Björkqvist et al., 1992). On another hand, fewer and closer friendship ties in females make indirect aggression more hurtful (Galen and Underwood, 1997). However, the significantly higher level of indirect aggression in females depends on the assessment methods, such as peer- or teacher-rating; in addition, the effect size of gender differences in indirect aggression is rather limited (Archer, 2004).

### Friendship Dynamics

Peers play a crucial role in shaping adolescents’ attitudes and behaviors by the presence of the selection and influence process (Brechwald and Prinstein, 2011). In the following subsections, the theory underlying the selection and influence process is reviewed. Afterward, the methodology to investigate the two processes is introduced.

#### Selection Process

As the saying “birds of a feather flock together” goes, youths actively seek out peers with a similar level of certain salient behavioral characteristics as friends (Werner and Crick, 2004; Brechwald and Prinstein, 2011). The interpersonal attraction theory argues that similarity among friends is positively related to attraction, regardless of attitudes, personality traits or behavioral actions (Byrne and Griffitt, 1973). People tend to feel more at ease and are more comfortable when associating with individuals who are similar to themselves; thus, they have the highest potential to establish friendships (Dishion et al., 1994). The similarity makes it easier for individuals to share feelings and to develop a sense of belonging, reducing conflicts as well as increasing trustworthiness (Veenstra et al., 2013). Furthermore, there are more opportunities to meet or affiliate with similar friends (Osgood and Anderson, 2004). For example, a drinker is more likely to meet other drinkers in a pub. These arguments all support the similarity-selection process.

#### Influence Process

Another process that accounts for similarity among friends is the influence process. Bandura’s (1977) social learning theory states that new behaviors can be acquired through observation and imitation in a social context, which is influenced by reinforcements such as rewards (Skinner, 1953). Taking deviancy training as an example, it is likely that teenagers will emulate the deviant behaviors of their close friends, especially when friends give verbal or non-verbal reinforcements for such behaviors (Dishion et al., 2001). Although socialization is unlikely to reinforce some behaviors (e.g., depression) by the population, it may provide unique benefits to close friendships (Rose et al., 2007). Some other theories, such as social comparisons and social norms, also explain the peer influence process. When an individual affiliates with a group, there is a fundamental need to compare with other group members and find the discrepancy. Individuals may make changes to reduce discrepancies (Festinger, 1954), resulting in stronger group homogeneity. This conformity may also be accounted by the normative influence process (Kruglanski and Webster, 1991). Adolescents adhere to the group norm for two main reasons: (1) aligning with group members, acquiring acceptance from them, and strengthening group identity by following how group members behave; and (2) avoiding awkwardness, embarrassment, and rejection by ceasing to engage in behaviors that deviate from the norm (Prinstein and Dodge, 2008; Shi and Xie, 2012).

#### Social Network Analysis

A longitudinal design with individual’s properties (e.g., aggression) and relationships with other individuals (social network) is necessary to investigate the homophily theory. How to analyze such network data is crucial when making firm statements of the two processes. In the existing longitudinal studies, researchers have adopted the contingency table approach (Kandel, 1978), the aggregated personal network approach (Kirke, 2004), and the structural equation modeling approach (Werner and Crick, 2004) for data analyses. These approaches follow a similar two-stage procedure, which aggregate the network data in the first stage and then analyze the summarized statistics in the second stage. However, the first stage seems arbitrary and the second stage violates the interdependence structure of the data (Steglich et al., 2010). In addition, the studies using these methods often ignore the dynamic nature of peer relationships and fail to control for network structure effects. Such limitations restrict the persuasion of similarity among friends (Steglich et al., 2010; Veenstra et al., 2013) and even distort the estimates of the selection and influence effects.

To overcome the drawbacks, the stochastic actor-based model at the complete social network level was developed (Snijders et al., 2010), which is implemented in the *Simulation Investigation for Empirical Network Analyses* software (SIENA). Such a model aims to represent network dynamics based on longitudinal data and examine the factors that drive friendship dynamics (Snijders et al., 2010). The “actor-based” nature means that the evolution of the network is organized by individual actors who can create, maintain, and terminate ties to other individuals (Ripley et al., 2016). Using SIENA, researchers can both disentangle the selection effects from influence effects and concurrently model network evolution and behavioral changes (Steglich et al., 2010).

To the best of our knowledge, there have been fewer than ten published papers concerning friendship dynamics on aggression using this method (see Table 1). These empirical findings, however, were not fully consistent because of varied measurements, forms of aggression, samples from various cultures, and so on. The following sections summarize what the existing studies of the association between friendship dynamics and aggression have yielded thus far. In general, significant influence effects were found in the majority of the SIENA studies, whereas the evidence for the select effects was only found in half of the studies. However, the moderating role of gender is a lack of investigation in most of the studies.

**TABLE 1:** Studies of aggression using the social network analysis method.

				**Selection process**		**Influence process**	
					
**Study**	**Nation**	**Aggression form**	**Assessment**	**Aggressive attraction**	**Selection effects**	**Influence Influence effects**	**Gender’s moderation**
Laninga-Wijnen et al., 2017	Dutch	Overall	Peer	Partly Sig.^a^	Partly Sig.^b^	Sig.	NA
Molano et al., 2013	United States	Overall	Teacher	NA	Non-sig.	Sig.	NA
Logis et al., 2013	United States	Overall	Peer	Negative Sig.	Non-sig.	Sig.	NA
Sijtsema et al., 2010	United States	Direct and Indirect	Self	Non-sig. (direct and indirect)	Non-sig. (direct and indirect)	Non-sig. (direct) Sig. (indirect)	NA
Dijkstra et al., 2011	Chile	Physical and Indirect	Peer	Non-sig. (physical and indirect)	Partly Sig.^c^ (physical and indirect)	Non-sig. (physical) Sig. (indirect)	NA
Dijkstra and Berger, 2018	Chile	Physical	Peer	Non-sig.	Non-sig.	Non-sig.	Non-sig.
Rulison et al., 2013	United States	Physical	Peer	Positive Sig.	Sig.	Sig.	Non-sig.
Shin, 2017	South Korea	Physical	Teacher	Positive Sig.	Sig.	Sig.	Stronger for boys
Kornienko et al., 2018	United States	Physical	Self	Non-sig.	Partly Sig.^d^	Sig.	NA

*Peer = peer nomination. Teacher = teacher reports. Self = self reports. Sig. = significant. Non-sig = non-significant. NA = the study did not add the effect to the model. ^*a*^The preference for aggressive classmates was only significant in classes with moderate popularity norms for aggression.^*b*^The selection effect was only significant in classes with high popularity norms for aggression.^*c*^When taking gender into consideration, the significant selection effects for physical and indirect aggression were absent. ^*d*^The selection effect was only significant in younger cohort (6th grade) but not in older cohort (7th grade).*

### Aggression and Friendship Dynamics

#### Aggression and Selection Process

Although aggression is not encouraged generally, aggressive adolescents still received friendship nominations from similarly aggressive peers and from a broader spectrum of teenagers (Rodkin et al., 2006). The former is corresponding to the selection effects, while the latter is corresponding to the attraction effects. The two effects are normally included in social network analysis.

##### Attraction effects of aggressive classmates

Research has found that adolescents regard aggressive peers popular and cool (Graham and Juvonen, 2002; Rodkin et al., 2006). However, research using SIENA which controlled other effects (e.g., similar-aggressive and same-gender preference) shows mixed results in different forms of aggression. Firstly, a significant preference for physically aggressive classmates was found in two studies (Rulison et al., 2013; Shin, 2017). It is explained that physical aggression is an approach both to achieving, enhancing, and maintaining high status among peers (Cillessen and Mayeux, 2004) and to attaining dominance (Pellegrini and Long, 2002). However, such a preference for physically aggressive peers is absent in samples from Chile (Dijkstra et al., 2011; Dijkstra and Berger, 2018) and the United States (Kornienko et al., 2018). Secondly, with regard to verbal aggression, a large-scale survey reported that having more friends is positively associated with verbal aggression (Wang et al., 2009). However, there is a lack of literature from Table 1 that specifically addressed the association between verbal aggression and friendship dynamics. One study combining verbal aggression with physical aggression found that there were no differences in receiving friend nominations among different levels of directly aggressive students (Sijtsema et al., 2010). In contrast, research on the attraction of indirectly aggressive adolescents is more consistent. There was no preference for choosing classmates who have a high- or low- level of indirect aggression as friends (Sijtsema et al., 2010; Dijkstra et al., 2011).

Some research did not distinguish any forms of aggression, showing completely different results – positive (Laninga-Wijnen et al., 2017), negative (Logis et al., 2013), and non-significant (Molano et al., 2013) associations between individuals’ overall aggression and the friendship nominations from others. Since attraction effects seem varied among the forms of aggression, it would be better to explore them individually.

##### Selection effects of aggressive classmates

In line with the interpersonal attraction theory (Byrne and Griffitt, 1973), some empirical studies demonstrated that aggressive youths make friends with peers who display similar levels of aggressive behaviors (e.g., Pellegrini et al., 1999; Werner and Crick, 2004). However, the studies using SIENA show a different picture with regard to subtypes of aggression.

Through the stochastic actor-based modeling, the majority of the studies reported significant selection effects regarding physical aggression (e.g., Dijkstra et al., 2011; Rulison et al., 2013; Shin, 2017). However, two studies found that this effect was absent when taking gender into consideration, which means that being the same gender is a more important determinant of friendship formation compared with having similar levels of physical aggression (Dijkstra et al., 2011; Dijkstra and Berger, 2018). Hence, when investigating the selection effects, it is necessary to control for the same-gender preference.

Again, little is known regarding whether the selection effect exists in verbal aggression specifically. Studies integrating verbal and physical aggression found that there is no preference to affiliate with peers who are similar in direct aggression with themselves after considering the same-gender preference (Sijtsema et al., 2010; Dijkstra et al., 2011). Although research has found that verbal aggression is a negative predictor of peer acceptance (Chang et al., 2005), whether verbally aggressive adolescents tend to make friends with each other remains unclear.

With respect to indirect aggression, researchers reported that the selection effect was not significant after including gender in the model (Sijtsema et al., 2010; Dijkstra et al., 2011). However, research on indirect aggression and friendship dynamics is rather limited as well, which require more samples to provide confirm conclusion. When combing all the forms of aggression together, the selection effect for similar aggressive peers was absent in general (e.g., Logis et al., 2013; Molano et al., 2013).

#### Aggression and Influence Processes

Another potential mechanism for the homophily theory is due to the influence process. After controlling for the selection process, research has found friends’ aggression contributes to shaping adolescent aggression (e.g., Logis et al., 2013; Molano et al., 2013; Laninga-Wijnen et al., 2017). In addition, much theory argues that gender plays a crucial role in adolescents’ behavioral development (Rose and Rudolph, 2006). Whether boys and girls show differences in the influence process of aggression was investigated initially but showed somewhat different results (Rulison et al., 2013; Shin, 2017; Dijkstra and Berger, 2018).

##### Influence effects of aggressive friends

Aggression seems infectious within peer groups. Espelage et al. (2003) found remarkable effects of peer groups on influencing adolescents’ fighting (physical aggression) and bullying (verbal and indirect aggression). Similarly, research based on social network analysis also has confirmed the influence effect in overall aggression with one accord (Logis et al., 2013; Molano et al., 2013; Laninga-Wijnen et al., 2017). However, specific to physical aggression, the research results are less consistent. The influence effects of physical aggression were observed in three studies (Rulison et al., 2013; Shin, 2017; Kornienko et al., 2018), but absent in two studies (Dijkstra et al., 2011; Dijkstra and Berger, 2018). When integrating verbal and physical aggression, this effect is non-significant (Sijtsema et al., 2010).

Compared with direct forms of aggression, the influence process is more common for indirect aggression. Friendships provide potential contexts for indirect aggression (Kawabata et al., 2012) because such aggression (e.g., spreading rumors through social networks) depends particularly on social networks. Empirical studies have provided evidence for this contention. Espelage et al. (2003) found that peer contexts explained more variance in non-physical aggression. Youths’ indirect aggression was positively predicted by indirectly aggressive friends (Werner and Crick, 2004). At the social network level, researchers also reported significant influence effects for indirect aggression (Sijtsema et al., 2010; Dijkstra et al., 2011).

##### Moderating role of gender on the influence process

Gender is a powerful organizer of peer friendships throughout adolescent development (Poulin and Pedersen, 2007) and is often examined as a typical characteristic of friendships (Mehta and Strough, 2009). It has been shown that the structure and features of networks differ between boys and girls. For example, girls’ networks are smaller and more intimate (Low and Espelage, 2013). That is, girls prefer to make a few best friends (vs. boys’ relationships center more around large group activities like playing basketball games) and tend to stress more emotional intimacy (Maccoby, 1990) by spending more time in relationship activities (Perry and Pauletti, 2011) and being more relationship-oriented than boys (Su et al., 2009). Girls’ greater emotional investments and group cohesion make friends’ behaviors especially influential for them and suggest more importance in adhering to group norms (Haynie et al., 2014). To maintain connections with their best friends, girls are more willing to follow friends’ behavior patterns and influenced more greatly by friends. In contrast, boys are more “things oriented” and devote more time to playing computer games, watching televisions, working with tools and other individual activities (Perry and Pauletti, 2011). They reported a lower level of intimacy and self-disclosure in friendships than girls did (Camarena et al., 1990). Additionally, friendships are greatly gender-segregated in early adolescence (Ruble et al., 2006). Same-gender peers play a key role in the socialization of stereotypical gender roles (Mehta and Strough, 2009). Thus, there is a reason to presume that the influence process may differ across boys and girls, especially for strongly gender-segregated countries such as China, where children regard opposite-gender interactions less favorably than their American counterparts (Li et al., 2012).

With respect to the friendship dynamics of physical aggression, studies that examined gender’s moderating role in the influence process drew different conclusions. Rulison et al. (2013) assumed that girls were more susceptible to being influenced by their friends’ physical aggression, although the result was non-significant. This expectation was motivated by the “normative experience” hypothesis (Hanish et al., 2005). That is, it is non-normative for girls to be exposed to externalizing peers (e.g., aggressive friends), making the relationships with externalizing peers more salient and potentially more influential (Hanish et al., 2005). Consistent with the hypothesis, the authors found that the peer socialization effects of externalizing problems were significant for young girls but not for boys (Hanish et al., 2005). Similarly, another study found that girls were more susceptible to being influenced by the violence of their friends (Haynie et al., 2014). Shin (2017), however, reported that the influence of friends on physical aggression was stronger for boys. Their argument was that boys are more likely to emphasize dominance, hierarchies and interpersonal status and to use more physical aggression to influence others (Dawes and Xie, 2014). Dijkstra and Berger (2018) found the influence process in physical aggression did not differ in all-male, all-female, and mixed-sex classes.

Regarding other forms of aggression, there is a lack of SIENA research on the moderating role in the influence process of verbal or indirect aggression. Reviewing studies using other methods, it seems that girls are more vulnerable by friends’ non-physical aggression. When a friend speaks ill of others, girls are more likely to participate, as they are afraid of becoming the next target to suffer exclusion or rumors (Werner and Crick, 2004). Additionally, girls tend to feel more empathy and to show reciprocity toward friends (Rose and Rudolph, 2006), which increases their probability of adopting friends’ behaviors. Moreover, girls develop verbal and social skills earlier and more proficiently than boys (Björkqvist et al., 1992). That is, it seems easier for girls to learn verbal and indirect aggressive strategies from their friends. It was evidenced that the indirect aggression of friends only predicted girls’ subsequent indirect aggression (Werner and Crick, 2004).

### Chinese Cultural Influences

The above-described conclusions were mostly drawn from studies conducted in the West. However, teenagers’ group behaviors should be understood within the contexts in which they occur (Farver, 1999; Xu et al., 2003, 2004). Culture plays an important role in perceiving and shaping aggressive behaviors (Bergeron and Schneider, 2005). Hence, great caution should be thrown when generalizing the conclusions from Western culture to other cultures.

Chinese culture is of interest here is due to its particular emphasis on relationships and harmony, which indicates potential differences compared with the Western culture. In detail, Chinese culture is typically collectivistic, which is characterized by an emphasis on close interdependence of relationships and a sense of obligation to the group, while the individualistic culture in the West values personal goals and a sense of obligation to the individual (Forbes et al., 2009). Moreover, one striking difference between China and the West is Confucian work dynamism, such as an emphasis on harmony and social order, which is in conflict with aggression (Chinese Culture and Connection, 1987; Forbes et al., 2011).

#### Chinese Culture in the Selection Process

Research has revealed that collectivism is negatively related to adolescents’ aggression (Forbes et al., 2009; Li et al., 2012). In contrast to the studies that found aggressive adolescents are regarded popular in Western samples (Graham and Juvonen, 2002; Rodkin et al., 2006), studies found that physically and indirectly aggressive students had lower peer status (e.g., less acceptance, more rejection, and less perceived popularity) in Taiwan (Tseng et al., 2013).

The three forms of aggression are treated differently in the Chinese context. Physical aggression, deviating from Chinese social norms, is strongly inhibited by both teachers and parents (Lu et al., 2018) and is negatively associated with popularity among Chinese adolescents (Tseng et al., 2013; Owens et al., 2014). Hence, the preference for selecting physically aggressive peers as friends might be absent in the Chinese context. Verbal aggression, however, is less strictly forbidden as physical aggression in China. Students in Chinese junior high schools used verbal aggression most commonly, followed by indirect aggression and then physical aggression (Jiang, 2017). Chinese subjects even exhibit more frequent verbal aggression than their counterparts in the U. S (Niem and Collard, 1972). That is, verbal aggression might be a more common approach to deal with conflict in China. Indirect aggression generates stressful relationships, which is particularly detrimental to group functioning and harmony in the Chinese culture compared with the Western culture (Kawabata et al., 2012). It has been found that indirect aggression was negatively associated with popularity among Chinese adolescents (Tseng et al., 2013; Owens et al., 2014). Hence, the preference for indirectly aggressive peers is much less likely.

With regard to the similarity-selection effects, it might be stronger in Chinese samples. Aggressive teenagers generally experience both punishments from teachers and parents and rejections from peers in China (Xu et al., 2003). It is especially hard for such teenagers to form positive peer relationships; however, they may organize with other aggressive adolescents to establish a support network (Xu et al., 2004; Chen et al., 2008).

#### Chinese Culture in the Influence Process

Despite the Chinese social norm for harmony, once adolescents aligned with aggressive peers, reinforcements or norms within their specific contexts may play a more salient role in peer influence processes (Rose et al., 2007). Moreover, Chinese emphasis on sensitivity to others might strengthen the power of peer influence. Since peer influence seems more important for non-physical (i.e., verbal and indirect) aggression (Espelage et al., 2003), in addition to more negative reinforcements for physical aggressive behaviors in China, the influence effects of physical aggression might not as strong as the other two subtypes of aggression.

## Present Study

Although the research on aggression and friendship dynamics has been carried out, extending our understanding of the relationships between social networks and adolescents’ aggression, there remain conflicting conclusions and unclear insight into this topic. In this section, we summarize the main limitations, state our intention of overcoming such limitations in the current study, and then illustrate our research questions as well as hypotheses.

### Potential Limitations in the Existing Literature

One of the limitations in the existing studies is that verbal aggression has not received sufficient attention in the literature (Poling et al., 2019). Early studies primarily concentrated on physical aggression or direct aggression, especially boys’ physical aggression; gradually, interest in indirect aggression has increased. As a specific oral expression of aggressive behavior, verbal aggression should be distinguished from physical aggression (Chang et al., 2005). In addition, verbal aggression is the most prevalent form of aggression (Wang et al., 2009; Jiang, 2017) and has specific impacts on cognitive reactivity (Cole et al., 2014); thus, researchers should commit more resources to discover the underlying process of verbal aggression (Poling et al., 2019). Few studies have focused on the relationship between verbal aggression and friendship dynamics, which means that friendship dynamics in verbal aggression is less well understood. To address this question, the current study intends to extend social network studies on peers and verbal aggression. Similarly, whether gender plays a moderating role in the influence process of verbal and indirect aggression remains unclear, which is also the question we aim to figure out.

Furthermore, these related studies were primarily conducted in Western culture (Gallupe et al., 2018), except one study conducted in Korea (Shin, 2017), reflecting insufficient samples from Eastern culture. In this research, we focused on the association between social networks and aggression in Chinese settings for the following reasons. Firstly, Chinese children tend to “see the world as a network of relationships” (Hsu, 1981), which is consistent with the collectivistic culture and Confucian dynamic in China. Hence, they make more efforts to avoid conflicts, make more agreements and maintain more relationships than their counterparts in the West (Woan et al., 2001). Secondly, a Chinese sample represents a more suitable sample when using the stochastic actor-based model. One primary limitation of previous research studies is that friend nominations are limited to within a class, and therefore important friendship ties that exist outside of class or school may be missed (e.g., Laninga-Wijnen et al., 2017; Shin, 2017). Classes in many Western countries are flexible. Students often move to different classes, mixing with different students. In contrast, Chinese middle schools have fixed classes, and students stay in the same class and affiliate with the same classmates for years (Niu et al., 2016). Therefore, the within-class peer nomination is more appropriate to capture the social networks of adolescents in China. Furthermore, many Chinese middle schools offer morning and afternoon individual study classes, which extends school time and provides more opportunities for students to develop their friendships. It is evidenced that the time spent with delinquent peers greatly predicts self-delinquency (Agnew, 1991).

### Research Questions and Hypotheses

In a nutshell, we aim to address four questions regarding physical, verbal, and indirect aggression in the Chinese context: (RQ1) Do adolescents nominate aggressive peers as friends? (RQ2) Do adolescents tend to make friends with peers who have a similar level of aggression? (RQ3) Does friends’ aggression influence adolescents’ aggression? (RQ4) Are there any gender differences regarding the influence process of aggression?

Our hypotheses are as follows. For RQ1, we hypothesize that Chinese students would not tend to select physically (H1a), verbally (H1b), or indirectly (H1c) aggressive classmates as friends, but our confidence of H1b is lower because verbal aggression is not strongly unfavorable in China (Niem and Collard, 1972). For RQ2, due to more chances to be exposed to similarly aggressive peers, we expect that Chinese adolescents tend to nominate classmates who have a similar level of physical (H2a), verbal (H2b), or indirect (H2c) aggression as friends generally. For RQ3, it is assumed that the peer influence process is also a mechanism for the homogeneity of the three subtypes of aggression (H3a, H3b, and H3c correspond to physical, verbal and indirect aggression, respectively) among friends in China, but our confidence for H3a is lower due to the particular prohibition of physical aggression. For RQ4, we hypothesize girls were more vulnerable to being influenced by friends’ physical aggression (H4a) due to the “normative experience” pattern (Hanish et al., 2005). Similarly, this study hypothesizes that friendships are especially likely to influence girls’ verbal (H4b) and indirect (H4c) aggression due to girls’ special emphasis on relationships (Haynie et al., 2014).

## Materials and Methods

### Procedures

The study was reviewed and approved by the research ethics committee at the corresponding author’s institution. Permission forms were sent to parents, all of whom permitted their children to participate in the study. The longitudinal data were collected at three time points: June 2015 (spring term of 7th grade), December 2015 (fall term of 8th grade), and June 2016 (spring term of 8th grade), respectively.

### Participants

There were 1354 students participated in wave 1 (54% males, mean age = 13.55 years old), 1332 students in wave 2 (53% males), and 1266 students in wave 3 (53% males). The students were enrolled in 25 classrooms (mean classroom size = 52.7) across four junior high schools located in suburban and rural communities in Central China. From wave 1 to wave 2, 27 students joined the classes and 49 students either transferred to another school or were absent on the day of data collection; from wave 2 to wave 3, the corresponding numbers were 3 and 70. Twenty-five percent of the participants’ parents finished senior high school education, and more than 80% of the parents completed junior high school education. More than 57% of the participants had an approximate annual household income of between 6,000 and 50,000 RMB, which is representative of Chinese citizens’ income, according to the National Bureau of Statistics of China.

### Measures

#### Friendship Networks

Class rosters were provided to each student, and the students were told to write up to five names of their best friends within the classroom (Dijkstra et al., 2011; Dijkstra and Berger, 2018). It is more accurate to refer to “best friends” rather than “friends” because in the Chinese context, “friends” are literally people you know, not close and intimate peers (Xu et al., 2004). On average, the students nominated 3.11, 2.75, and 2.66 names in wave 1–3, respectively. The social network analysis used ones or zeros to indicate the presence or absence of friendship ties, which constitute an adjacency matrix for each class. The joiners and leavers who join or leave the class between observations (e.g., transfer students) were denoted by structural zeros and non-response missing data were handled through SIENA (Ripley et al., 2016).

#### Subtypes of Aggression

We adapted the Direct and Indirect Aggression Scales (DIAS; Björkqvist et al., 1992) into a peer nomination questionnaire. This measure was translated into Chinese and then back-translated into English by two graduates majoring in English. The students were told to nominate at most five classmates who best fit the item separately. The adapted scale included nine items in total with three items for each subtype. The physical aggression dimension contained “he (she) always makes troubles and fights with others,” “he (she) often kicks and pushes others,” and “he (she) often breaks others’ stuff on purpose.” The Cronbach’s alpha values were 0.91, 0.93, and 0.93 for wave 1–3, respectively. The verbal aggression dimension contained “he (she) always loses his (her) temper and quarrels with others,” “he (she) likes to make fun of others,” and “he (she) always verbally threatens and intimidates others.” The Cronbach’s alpha values were 0.88, 0.90, and 0.90 for wave 1 to 3, respectively. The indirect aggression dimension contained “he (she) likes to speak ill of others behind their backs,” “he (she) likes to deliberately isolate certain classmates to make them feel bad” and “he (she) often sows discord between students.” The Cronbach’s alpha values were 0.92, 0.93, and 0.92 for wave 1–3, respectively.

For each subtype of aggression, the nominations were totaled and divided by the number of possible nominators to eliminate the impact of classroom size. Because the dependent behavioral variables are required to be non-negative integers in SIENA (Ripley et al., 2016), we transformed the original percentages into three almost equally populated categories, referring to Laninga-Wijnen et al. (2017). More specifically, we placed the original proportional scores in descending within every school and recoded the top 33% as 2 (high), the bottom 33% as 0 (low), and the rest as 1 (medium). In general, lowly aggressive individuals barely got nominations in the DIAS, and highly aggressive ones received more than 3% of the nominations. Through this method, the distribution of the data was relatively balanced among each category.

### Analysis Strategy

The data were analyzed using the SIENA package in R 3.3.1 software. The estimates were derived from Markov Chain Monte Carlo (MCMC) iterative simulations. We estimated each network within each class separately and then combined them in a meta-analysis using *siena08* (Snijders and Baerveldt, 2003).

Firstly, three structural network effects were controlled: the *density* effect, the *reciprocity* effect, and the *transitive triplets*. *Density* describes the overall tendency to nominate classmates as best friends. A positive *Density* value indicates an increasing likelihood of friendship ties over time. *Reciprocity* and *Transitive triplets* describe the tendency to reciprocate friendship nominations and to affiliate with friends’ friends, respectively. It is necessary to control the structural network effects. For example, if two aggressive adolescents form their friendship due to a shared friend, ignoring the *transitive triplets* may overestimate the selection effect of similar aggressive behaviors, which further affects the estimates of influence effects (Steglich et al., 2010).

Secondly, selection processes were estimated. The *ego* and *alter* effect implies the extent to which a certain individual characteristic was related to giving and receiving nominations. *Similarity* effects describe the extent to which students nominated best friends who were similar to themselves with respect to a particular characteristic. In the current study, the effect of making friends with same-gender peers (*gender similarity*) was controlled because teenagers tend to make friends with peers of the same gender (Benenson et al., 1998). Omitting this potential preference for same-gender classmates may distort the selection effects for aggression similarity.

Thirdly, we focused on the influence processes. The basic distributional features (shape effects) of aggression across the three waves were included in this analysis, including the *linear* and *quadratic shape* effects. The purpose is to control the developmental trajectories of aggressive behavior and to offer a more reliable inference for the influence process. Specifically, the *linear effect* is an average tendency toward a decrease or increase in aggression, and the *quadratic effect* reflects the feedback effect of behavior on itself (Snijders et al., 2010). *The effect from gender* was taken into consideration to control the main effect of gender on behavior changes. The peer influence effect is expressed as *average similarity*, which reflects the preference of adolescents to become similar with respect to the behavior of their nominated friends on average. The moderating effects were examined by adding the interaction between the actors’ *average similarity* and *gender ego*. A positive coefficient for the interaction indicated that boys were more susceptible to being influenced by aggressive best friends when boys were dummy recoded as 1 and girls as 0.

## Results

The analysis results are organized into two parts: (1) descriptive statistics, and (2) social network analyses.

### Descriptive Statistics

Table 2 presents the description of the sample, the variables at each observation measurement and the longitudinal changes. Average ties, average outdegree (the average number of giving nominations), and density decreased over the three time points, indicating that the participants were less likely to nominate best friend ties over time. The Jaccard index reflects the Jaccard distance between successive networks, indicating the stability between networks. Jaccard values of 0.30 and higher are good; values lower than 0.20 indicate that stability might be difficult to estimate; and values lower than 0.10 are poor (Ripley et al., 2016). In this study, the Jaccard values were 0.22 and 0.25, which were acceptable. Table 2 also presents peer-nominated aggression from wave 1 to wave 3. Boys received more nominations than girls in all subtypes of aggression. This result was particularly revealing for physical aggression; the boys’ score was more than twice the value of the girls’ score. The differences between the genders narrowed for indirect aggression.

**TABLE 2:** Descriptive statistics for best friends network and aggressions across waves.

	**Wave 1**	**Wave 2**	**Wave 3**		**Wave 1-2**	**Wave 2-3**
***Friendship networks***				Average number of ties dissolved	115.64	90.64
*Participants, N*	1354	1332	1266	Average number of ties emerged	95.76	85.92
Average ties	174.44	154.48	143.16	Average number of ties maintained	58.60	56.96
Average outdegree	3.11	2.75	2.66	Jaccard index	0.22	0.25
Density	0.06	0.05	0.05			
***Physical aggression***				***Physical aggression change percentage***		
Boys average (*SD*)	1.30 (0.77)	1.29 (0.77)	1.21 (0.81)	Increased (boys/girls)	17.10/17.08	13.22/20.26
Girls average (*SD*)	0.54 (0.67)	0.49 (0.64)	0.52 (0.68)	Decreased (boys/girls)	17.92/21.00	18.39/16.75
***Verbal aggression***				***Verbal aggression change percentage***		
Boys average (*SD*)	1.26 (0.73)	1.27 (0.71)	1.20 (0.78)	Increased (boys/girls)	17.78/12.85	15.09/18.82
Girls average (*SD*)	0.96 (0.72)	0.84 (0.73)	0.83 (0.75)	Decreased (boys/girls)	16.14/23.35	19.11/19.14
***Indirect aggression***				***Indirect aggression change percentage***		
Boys average (*SD*)	1.19 (0.73)	1.18 (0.73)	1.08 (0.77)	Increased (boys/girls)	20.38/16.61	13.65/17.86
Girls average (*SD*)	1.07 (0.73)	0.98 (0.73)	0.95 (0.75)	Decreased (boys/girls)	19.97/24.45	21.98/19.30

**FIGURE 1:**
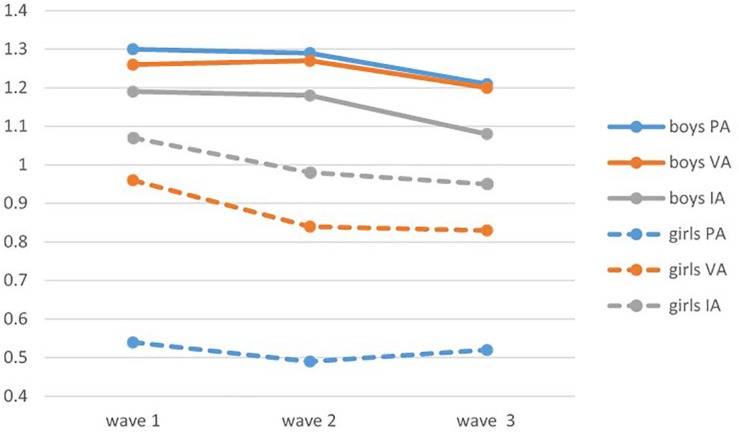
Developmental trajectories of physical, verbal and indirect aggression of boys and girls in the three waves. PA, physical aggression; VA, verbal aggression. IA, indirect aggression.

The developmental trajectories of the three subtypes of aggression are illustrated in Figure 1. Nearly all forms of aggression exhibited a tendency to decrease among both the boys and the girls, with the exception of girls’ physical aggression, which increased a bit from wave 2 to wave 3.

Correlations among the three subtypes of aggression and the number of best friend nominations received for both the boys (above the diagonal) and the girls (below the diagonal) are presented in Table 3. The correlations among the types of aggression during the three waves were all higher than 0.40, and most reached 0.80 for boys and 0.70 for girls. The number of best friend nominations received was positively correlated with the number received in the successive wave. In addition, for the girls, the number of best friend nominations received was negatively and mildly correlated with all types of aggression in all waves. For the boys, the correlations were much lower, and verbal aggression exhibited a non-significant correlation to the number of best friend nominations received.

**TABLE 3:** Correlations among aggressions and best friend nominations received across waves.

	**1**	**2**	**3**	**4**	**5**	**6**	**7**	**8**	**9**	**10**	**11**	**12**
(1) Physical-Wl	–	0.89**	0.80**	0.84**	0.80**	0.72**	0.81**	0.77**	0.66**	−0.06	−0.10**	−0.11**
(2) Verbal-Wl	0.84**	–	0.85**	0.74**	0.82**	0.72**	0.70**	0.77**	0.65**	−0.01	−0.05	−0.04
(3) Indirect-Wl	0.66**	0.78**	–	0.64**	0.70**	0.74**	0.65**	0.69**	0.71**	−0.11**	−0.13**	−0.11**
(4) Physical-W2	0.80**	0.80**	0.58**	–	0.91**	0.84**	0.91**	0.84**	0.75**	−0.06	−0.09**	−0.10**
(5) Verbal-W2	0.68*’	0.81**	0.69**	0.79**	–	0.88**	0.84**	0.88**	0.76**	−0.02	−0.05	−0.06
(6) Indirect-W2	0.53**	0.67**	0.80**	0.63**	0.81**	–	0.80**	0.81**	0.83**	−0.10**	−0.14**	−0.15**
(7) Physical-W3	0.58**	0.53**	0.40**	0.66**	0.52**	0.41**	–	0.92**	0.85**	−0.08**	−0.10**	−0.14**
(8) Verbal-W3	0.53**	0.68**	0.56**	0.61**	0.74**	0.62**	0.75**	–	0.89**	<−0.01	−0.04	−0.07
(9) Indirect-W3	0.47**	0.57**	0.72**	0.52**	0.60**	0.76**	0.58**	0.76**	–	−0.06	−0.08*	−0.12**
(10) Best friend-Wl	−0.16**	−0.20**	−0.21**	−0.13**	−0.15**	−0.17**	−0.15**	−0.14**	−0.13**	–	0.67**	0.60**
(11) Best friend-W2	−0.13**	−0.14**	−0.19**	−0.14**	−0.13**	−0.19**	−0.13**	−0.09**	−0.12**	0.58**	–	0.67**
(12) Best friend-W3	−0.18**	−0.18**	−0.18**	−0.16**	−0.14**	−0.15**	−0.18**	−0.16**	−0.15**	0.51**	0.65**	–

*p < 0.05, **p < 0.01. Correlations above the diagonal reflect boys, and correlations below the diagonal reflect girls. 1 = physical aggression in wave 1, 2 = verbal aggression in wave 1, 3 = indirect aggression in wave 1, 4 = physical aggression in wave 2, 5 = verbal aggression in wave 2, 6 = indirect aggression in wave 2, 7 = physical aggression in wave 3, 8 = verbal aggression in wave 3, 9 = indirect aggression in wave 3, 10 = the number of receiving best friend nomination in wave 1, 11 = the number of receiving best friend nomination in wave 2, and 12 = the number of receiving best friend nomination in wave 3.

### Social Network Analysis Results

#### Network Structure Results

The results associated with network structural effects are presented in Table 4. The students preferred mutual friendships (*reciprocity* estimate = 1.19, *p* < 0.001) and to be friends with their best friends’ friends (*transitive triplets* estimate = 0.29, *p* < 0.001). All of the structural effects were controlled when we examined the selection and influence processes.

**TABLE 4:** Meta-analyses of social network modeling of best friends and aggressions.

	***Est.***	***S.E.***	***Sig.***
**Structural effects**			
Density	−2.44	0.05	<0.001
Reciprocity	1.19	0.05	<0.001
Transitive triplets	0.29	0.01	<0.001
**Selection process**			
Gender alter	−0.10	0.06	0.113
Gender ego	0.10	0.05	0.046
Gender similarity	1.29	0.11	<0.001
Physical aggression alter	−0.05	0.04	0.145
Physical aggression ego	0.05	0.03	0.057
Physical aggression similarity	0.15	0.07	0.048
Verbal aggression alter	0.06	0.03	0.029
Verbal aggression ego	−0.01	0.02	0.749
Verbal aggression similarity	0.24	0.07	0.003
Indirect aggression alter	−0.09	0.03	0.003
Indirect aggression ego	<0.01	0.03	0.912
Indirect aggression similarity	0.19	0.08	0.020
**Influence process**			
Physical aggression: linear shape	−0.12	0.10	0.260
Physical aggression: quadratic shape	0.69	0.12	<0.001
Physical aggression: effect from gender	0.35	0.16	0.037
Physical aggression: average similarity	1.22	0.44	0.016
Physical aggression × gender ego	−1.70	0.60	0.025
Verbal aggression: linear shape	−0.10	0.08	0.253
Verbal aggression: quadratic shape	0.32	0.11	0.007
Verbal aggression: effect from gender	0.37	0.09	<0.001
Verbal aggression: average similarity	0.83	0.30	0.013
Verbal aggression × gender ego	−0.85	0.59	0.170
Indirect aggression: linear shape	−0.17	0.15	0.270
Indirect aggression: quadratic shape	0.49	0.33	0.151
Indirect aggression: effect from gender	0.14	0.10	0.170
Indirect aggression: average similarity	1.59	0.41	0.001
Indirect aggression × gender ego	0.41	0.54	0.458

#### Selection Process Results

Firstly, gender is a telling factor in friendship formation. Boys were more likely than girls to give best friend nominations (*gender ego* estimate = 0.10, *p* < 0.05), but they received the similar number of nominations as girls. The *gender similarity* estimate was 1.29 (*p* < 0.001), implying that the students tended to choose same-gender classmates as best friends.

Secondly, the results in *aggression alter* effects are the answers to RQ1 (whether aggressive adolescents attract friends). Students with higher levels of physical aggression did not differ from other students in giving and receiving best friend nominations, as the *physical aggression alter* and *ego* estimates were non-significant. Verbal and indirect aggression exhibited opposite pictures with respect to receiving nominations. Students with a higher level of verbal aggression were more likely to receive friend nominations (*verbal aggression alter* estimate = 0.06, *p* < 0.05). In contrast, students with a higher level of indirect aggression received fewer nominations (*indirect aggression alter* estimate = −0.09, *p* < 0.01). In sum, the results indicated that Chinese adolescents have a preference for verbally aggressive classmates (against H1b), but they do not tend to affiliate with indirectly aggressive classmates (support H1c). However, the preference for physically aggressive adolescents is absent (not support H1a).

There were significant selection effects for all of the subtypes of aggression (RQ2), as indicated by the positive *similarity* estimates. Namely, the students were more prone to initiate friendships with classmates who had a similar level of aggression. These results supported H2a, H2b, and H2c.

#### Influence Process Results

Firstly, the developmental changes in aggression were controlled. For physical and verbal aggression, the *linear shape effect* is non-significant, which implies a drift toward the midpoint of the range of physical or verbal aggression, while the significant *quadratic shape effect* can be regarded as a self-reinforcing effect (Ripley et al., 2016). This means that students with high levels of physical or verbal aggression were likely to exhibit higher levels of such aggression over time, whereas students with low levels of physical or verbal aggression were likely to exhibit further decreases over time. However, indirect aggression did not exhibit any linear or quadratic tendencies. The *effect from gender* in physical and verbal was significant, illustrating that boys and girls show different developmental trajectories in such aggression. This effect was controlled as well.

Secondly, the influence effects were significant for all the three subtypes of aggression, which supported H3a, H3b, and H3c. The students’ physical and verbal aggression was influenced by their best friends’ same type of aggression (*average similarity* for physical aggression = 1.22, *p* < 0.05; *average similarity* for verbal aggression = 0.83, *p* < 0.05). The only significant estimate of indirect aggression was *average similarity* (estimate = 1.59, *p* < 0.001), indicating that the students became increasingly similar to their best friends in terms of indirect aggression.

Thirdly, the results regarding the moderating effect of gender answer RQ4. Gender only moderates the influence process in physical aggression (*interaction effect* estimate = −1.70, *p* < 0.05), but not in verbal or indirect aggression. Girls were more susceptible to their best friends’ physical aggression than boys were. In other words, only H4a was confirmed.

## Discussion

In this study, we investigated the friendship dynamics of physical, verbal and indirect aggression in China using social network analysis (SIENA) and made interesting and culturally different findings.

### Attractive Verbally Aggressive Adolescents and Unattractive Indirectly Aggressive Adolescents

In the current study, we found that the three subtypes of aggression had varied associations with attraction. There was a preference for highly verbally aggressive peers and lowly indirectly aggressive ones, but no preference for physically aggressive classmates. Some previous studies have implied that in the West, physically aggressive youth are more attractive as friends (Rodkin et al., 2006; Rulison et al., 2013; Laninga-Wijnen et al., 2017) because they utilized physical aggression to attain dominance and social resource (Pellegrini and Long, 2002). However, the preference for these adolescents was absent in the Chinese context, which was in line with samples from Chile using peer nomination assessment for physical aggression (Dijkstra et al., 2011; Dijkstra and Berger, 2018). One potential explanation is that physically aggressive behavior is strictly prohibited by parents and teachers in China (Chen, 2010; Niu et al., 2016). When a student is criticized or punished by teachers in public, the individual is likely to feel ashamed and his or her reputation is impaired (loss of face); such a phenomenon is particularly salient in Chinese society (Qi, 2011). The positive effects of physical aggression on maintaining high status and showing physical strength might be offset by the negative effect of being punished, making the preference for physically aggressive adolescents non-significant in the Chinese context.

Unlike our hypothesis, Chinese adolescents prefer verbally aggressive classmates as best friends, which seems contrary to the Confucian work dynamism. This may be caused by Chinese participants’ “more sophisticated understanding of arguing” (Xie et al., 2015). Compared with the Western participants, Chinese respondents were more inclined to grasp the constructive potential of arguing, which was more than a verbal confrontation (Xie et al., 2015). Chinese students have higher verbal aggressiveness scores and are less avoidant of confrontation than their Western counterparts, indicating that Chinese were more motivated to participate in rather than avoid interpersonal argumentation (Niem and Collard, 1972; Xie et al., 2015). Additionally, because physical aggression is severely suppressed, verbal aggression becomes another powerful method for dealing with conflicts and acquiring high status in China. Thus, verbal aggression represents a relatively mild and constructive method for dealing with conflicts. Furthermore, the result might also be caused by variables (e.g., verbal ability and academic achievements) that we did not consider in this study, which could be addressed in the future.

The majority of SINEA studies found that students who were indirectly aggressive received equal friendship nominations as those who were not indirectly aggressive (Sijtsema et al., 2010; Dijkstra et al., 2011). In comparison, indirect aggression is considered much more unfavorable in China because it represents a potential threat to group harmony. In this study, adolescents who were high in indirect aggression received fewer best friend nominations, which confirmed our hypothesis. In other words, the negative effect of indirect aggression is much severer in the Chinese context.

### Selecting Similarly Aggressive Classmates as Best Friends

According to the selection hypothesis (Kandel, 1978), the similarity of aggression precedes the formation of friendships. Our results suggested that Chinese adolescents tended to initiate friendship with others who had similar levels of aggression after controlling for same-gender preference, which was consistent with some earlier research (Rulison et al., 2013; Shin, 2017; Laninga-Wijnen et al., 2017) and the interpersonal attraction theory (Byrne and Griffitt, 1973). Although several studies did not detect the evidence of selection based on aggression (Sijtsema et al., 2010; Logis et al., 2013; Molano et al., 2013), the selection effects of physical, verbal, and indirect aggression were all significant in our study. Since aggressive behaviors are actively discouraged in China, it is harder for them to make friends with non-aggressive ones. Thus, they turn to other aggressive peers to form a support system (Chen et al., 2008), confirming the selection hypothesis.

### Being Influenced by Best Friends’ Aggression

We found that adolescents tended to adopt friends’ aggressive behaviors over time, which was in line with the most previous studies (e.g., Logis et al., 2013; Rulison et al., 2013; Shin, 2017; Kornienko et al., 2018). The social learning theory (Bandura, 1977) could explain the influence process. Adolescents who have aggressive friends in school are faced with two conflicting modeling influences: one is from aggressive friends, while another is from teachers who prohibit aggressive behaviors. The former might be favored by youth because adult standards are relatively high and are not easy to meet (Bandura, 1977). In addition, from an operant conditioning perspective, peer approval (e.g., praise) can be regarded as a “generalized social reinforcer” (Skinner, 1953), which contributes to reinforcing the imitation of peer behavior. In other words, the advantages of adopting the group’s aggressive behaviors (e.g., reinforcement from friends and the sense of belonging to the group) over negative results such as punishment from teachers. Given their emphasis on relationships and obligation to groups (Forbes et al., 2009), it seems more important for Chinese adolescents to reduce discrepancies with group norms. Adding the current results to the existing literature, verbal aggression, physical aggression, and indirect aggression are consistently influential among friends in our study.

### The Moderating Role of Gender on Physical Aggression

Girls were more susceptible than boys to the effects of friends’ physical aggression, thus confirming the “normative experience” hypothesis; that is, non-normative friendship is potentially more influential (Hanish et al., 2005). It is well documented that “differential exposure” and “differential reaction” provide explanations for the gender gap in offending (Haynie et al., 2014). In detail, girls are much less exposed to risk factors such as aggressive friends, and when exposed, they are influenced more strongly. If a girl has best friends who often attack others, she may realize that it is not very uncommon to be physically aggressive and may adopt that type of behavior. Moreover, this greater influence might also be caused by girls’ more investment in friendships (Rose and Rudolph, 2006). Because China has greater gender segregation and a harmony culture, the “differential exposure and reaction” process and “normative experience” of physical aggression have been intensified. Thus, gender’s non-significant moderating role on the influence effect in the West (Rulison et al., 2013) became significant in our Chinese sample. Our result, however, was contrary to Shin’s (2017) study, which reported that boys are more likely to endorse dominance than girls and thus, physical aggression is more prominent among boys. Chinese adolescents prioritize harmony more than Korean participants (Zhang et al., 2005), and self-assertion and autonomy are not tolerated by Chinese parents (Solomon, 1972). Therefore, it does not make much sense to endorse dominance in relation to gender’s moderating role in China.

The influence effects of verbal and indirect aggression did not vary between genders. Verbal and indirect aggression between boys and girls did not differ as significantly as physical aggression, according to Table 2 and the meta-analysis where the gender difference regarding indirect aggression was not telling when the assessment consisted of peer nominations (Archer, 2004; Card et al., 2008). The “differential exposure” or “normative experience” hypothesis, therefore did not work in the current sample because it was equally normal for boys and girls to be verbally and indirectly aggressive. Future studies could reexamine the moderating role of gender on the influence process within Confucian work dynamism culture and within different countries to further investigate this effect.

### Limitations, Strengths, and Implications

Several limitations should be noted and possibly addressed in the future. Firstly, we used only the peer nominations method to assess aggression. Because different measurements could lead to different conclusions (Card et al., 2008), we cannot simply generalize the results to other situations, such as teacher- or parents- reports. Future studies could adopt other assessment methodologies to repeat the present study. Secondly, we limited the number of “best friend” nominations to five names. Many studies used unlimited friend nominations (Rulison et al., 2013; Laninga-Wijnen et al., 2017; Shin, 2017), except for Dijkstra et al. (2011), Dijkstra and Berger (2018). The primary influence of limited nominations is that structural network effects (e.g., reciprocity and transitivity) were likely to be underestimated (Dijkstra et al., 2011); even so, these effects were still significant in this study, indicating that the impact of limited nominations on structural effects might be mild. In addition, the average number of best friend nominations is fewer than 3.2 in this study, which indicated that most of the participants’ best friends were included. Thirdly, the current study only considered gender when assessing the influence process. Future studies could focus on other moderating variables (e.g., peer status, age, academic achievement) to investigate friendship dynamics and aggression more deeply.

This study has strengths. Firstly, the longitudinal design with a large sample size was adopted in our study, making it possible to discover the underlying mechanism of friendship and adolescent aggression. Secondly, we employed the stochastic actor-based model, which disentangles the selection and influence process, controls the developmental trajectory of aggression, and excludes the similarity effect of gender in addition to gender’s main effect, creating a potentially more powerful inference of the associations between aggression and friendship dynamics. Thirdly, we investigated the friendship dynamics among Chinese adolescents for the first time, extending the sample diversity. Chinese students spend much more time with classmates than Western students, so friendships within the class seem much more important to Chinese students. Fourthly, the current study contributes significantly to our understanding of one specific form of aggression – verbal aggression – by demonstrating its attraction to adolescents, significant selection and influence effects. The three subtypes of aggression have varied effects on friendship, indicating that researchers should distinguish different forms of aggression to gain a clearer insight into aggression research. Finally, the results of this study add to the literature on the moderating role of gender on physical aggression rather than verbal and indirect aggression.

Our findings have implications for prevention and intervention programs against aggression. For Chinese adolescents, verbal aggression is very frequent and relatively favorable. Hence, schools and parents should take measures to educate students that verbal aggression is as impolite and hurtful as physical aggression and attempt to reduce adolescents’ verbal aggression. Both physical aggression and verbal aggression have self-reinforce effects. Thus, students who have high levels of physical or verbal aggression should receive more supervision and guidance to prevent the situation from becoming more severe. The gender differences in physical aggression should alert educators that girls are more susceptible to adopting peers’ physically aggressive behavior. Once teachers find that girls have made physically aggressive friends, they should pay more attention to those girls.

## Data Availability Statement

The datasets generated for this study are available on request to the corresponding author.

## Ethics Statement

The studies involving human participants were reviewed and approved by Ethical Review Boards of Collaborative Innovation Center of Assessment for Basic Education Quality, Beijing Normal University. Written informed consent to participate in this study was provided by the participants’ legal guardian/next of kin.

## Author Contributions

MZ: study conception, data analysis, and manuscript writing and revision. HL: data analysis and manuscript revision. YZ: research materials, data collection, and manuscript revision.

## Conflict of Interest

The authors declare that the research was conducted in the absence of any commercial or financial relationships that could be construed as a potential conflict of interest. The reviewer CH declared a past collaboration with one of the authors HL to the handling Editor.
